# Are Cardiac Autonomic Nervous System Activity and Perceived Stress Related to Functional Somatic Symptoms in Adolescents? The TRAILS Study

**DOI:** 10.1371/journal.pone.0153318

**Published:** 2016-04-18

**Authors:** Karin A. M. Janssens, Harriëtte Riese, Arie M. Van Roon, Joke A. M. Hunfeld, Paul F. C. Groot, Albertine J. Oldehinkel, Judith G. M. Rosmalen

**Affiliations:** 1 University of Groningen, University Medical Center Groningen, Interdisciplinary Center for Psychopathology and Emotion regulation, Groningen, The Netherlands; 2 University of Groningen, University Medical Center Groningen, Department of Internal Medicine, Groningen, The Netherlands; 3 Erasmus Medical Center Rotterdam, Department of Medical Psychology & Psychotherapy, Rotterdam, The Netherlands; 4 Academic Medical Center, Department of Radiology, Amsterdam, The Netherlands; University of Miami School of Medicine, UNITED STATES

## Abstract

**Objective:**

Stressors have been related to medically insufficiently explained or functional somatic symptoms (FSS). However, the underlying mechanism of this association is largely unclear. In the current study, we examined whether FSS are associated with different perceived stress and cardiac autonomic nervous system (ANS) levels during a standardized stressful situation, and whether these associations are symptom-specific.

**Methods:**

We examined 715 adolescents (16.1 years, 51.3% girls) from the Dutch cohort study Tracking Adolescents’ Individual Lives Sample during the Groningen Social Stress Test (GSST). FSS were assessed by the Youth Self-Report, and clustered into a cluster of overtiredness, dizziness and musculoskeletal pain and a cluster of headache and gastrointestinal symptoms. Perceived stress levels (i.e. unpleasantness and arousal) were assessed by the Self-Assessment Manikin, and cardiac ANS activity by assessing heart rate variability (HRV-HF) and pre-ejection period (PEP). Perceived stress and cardiac ANS levels before, during, and after the GSST were studied as well as cardiac ANS reactivity. Linear regression analyses were used to examine the associations.

**Results:**

Perceived arousal levels during (beta = 0.09, p = 0.04) and after (beta = 0.07, p = 0.047) the GSST, and perceived unpleasantness levels before (beta = 0.07, p = 0.048) and during (beta = 0.12, p = 0.001) the GSST were related to FSS during the past couple of months. The association between perceived stress and FSS was stronger for the FSS cluster of overtiredness, dizziness and musculoskeletal pain than for the cluster of headache and gastrointestinal symptoms. Neither ANS activity levels before, during, and after the GSST, nor maximal HF-HRV and PEP reactivity were related to FSS.

**Conclusions:**

This study suggests that perceived stress levels during social stress are related to FSS, whereas cardiac ANS activity and reactivity are not related to FSS.

## Introduction

Functional somatic symptoms (FSS), like fatigue and chronic pain, are symptoms not fully explained by a conventional medical condition. They are common during adolescence and can have large impact on adolescents’ lives [1,2]. More insight into the developmental pathways of this important health problem might aid the development of effective prevention and intervention strategies. Stressors have often been suggested to play a prominent role in the development of FSS [3,4]. However, the mechanistic pathways of the association between stressors and FSS is largely unclear.

The most commonly examined pathway is that stressors result in high perceived stress levels and that these high perceived stress levels lead to feelings of tiredness and other somatic discomfort. High perceived stress levels have repeatedly been associated with FSS in adolescents [5–9]. Moreover, it has recently been found that high perceived stress levels indeed impair somatic recovery from physiological exhausting situations [9]. It is, however, unclear whether high perceived stress levels reflect anticipation stress, stronger psychological reactions during stressful situations or problems with psychological recovery, and thus sustained high perceived stress levels, after a stressful situation occurred.

Apart from maladaptive psychological responses to stress, also maladaptive physiological responses have been thought proposed to play a role in the relationship between stressful experiences and development of FSS. Specifically, inadequate reactions of the cardiac autonomic nervous system (ANS) system are thought to explain part of the association between stressors and FSS [10]. The cardiac ANS consists of two branches, namely the parasympathetic (PNS) and sympathetic nervous system (SNS) branch. The PNS is mainly responsible for maintaining normal growth and restoration of internal organs, and the SNS for redistributing metabolic output in times of external threat. Stress reactions are generally characterized by withdrawal of the PNS and heightened SNS activation. Since FSS are believed to result from adaptation to chronic stress, low PNS and high SNS levels have specifically been suggested to be related to FSS. However, research on the association between ANS functioning and FSS in adolescents and adults is scarce and results are conflicting [10–18]. Particularly the SNS has hardly been studied in relationship to FSS. Moreover, most previous studies have examined ANS activity during rest, and hence it is unknown whether FSS are stronger related to ANS activity before, during or after a stressful situation [19].

Another remaining question is whether psychological or physiological stress levels are particularly related to specific clusters of FSS. Much debate is going on in the field of psychosomatic medicine about whether all FSS result from the same underlying etiology and can thus be examined together (the lumping approach) or whether each FSS has its own specific background and should be examined separately (the splitting approach). Taking both approaches will shed more light on these different points of view. Previous research suggests that the association between FSS and cortisol responses may be symptom-specific. For example, fatigue and musculoskeletal pain symptoms have been found to be specifically related to morning cortisol levels [20,21], so to cortisol functioning not linked to a stressful situation. Headache and gastrointestinal symptoms have been found to be related to cortisol responses during stress and not to morning cortisol levels [20]. In the current study we want to examine whether specific FSS clusters are also stronger related to high perceived stress levels and ANS activity before, during and after a stressful situation.

Most previous research on the association between cardiac ANS and FSS examined absolute ANS activity levels. However, the use of absolute ANS activity levels has been criticized, since using these absolute ANS levels does not allow taking interindividual cardiac differences, for instance cardiac preload and afterload, into account [22]. Therefore, the use of cardiac ANS reactivity measures has been recommended [22]. Cardiac reactivity measures reflect within-person changes in ANS activity levels during different conditions. Cardiac reactivity measures thus allow us to examine within-subject changes in ANS activity in a between-subject design. In line with reasoning about ANS levels, high cardiac ANS reactivity measures are expected to be related to FSS, but it is currently unknown whether ANS levels and ANS reactivity levels are both related to FSS.

The aim of the current study is to examine whether perceived stress levels and ANS activity before, during, and after a standardized stressful situation are related to FSS in adolescents. We hypothesize that FSS are associated with high perceived stress levels, low PNS levels and high SNS levels before, during and after a standardized stressful situation and that these associations are symptom-specific. Further, we hypothesize that FSS are related to high cardiac ANS reactivity. Our hypotheses were tested in a large population-based sample of adolescents (*N* = 715) who participated in a social stress test.

## Methods

### Participants

The data were collected in a subsample of Tracking Adolescents’ Individual Lives Survey, a large prospective population study of Dutch adolescents with bi- or triennial measurements from age 11 to at least age 25. Thus far, five assessment waves have been completed. During T1, 2230 children were enrolled in the study (for more details about the sample selection, see [23]), of whom 1816 (81.4%) participated in T3. During T3, 744 adolescents were invited to perform an additional experimental session; 715 (96.1%) agreed to do so. Adolescents with a high risk of mental health problems had a greater chance of being selected for the experimental session, since mental health was the main focus of the Tracking Adolescents’ Individual Lives Survey. High risk was defined based on three criteria: temperament (i.e., high frustration and fearfulness and low effortful control); lifetime parental psychopathology; and living in a single-parent family (see also [24]). In total, 66.0% of the focus sample had at least one of the above-described risk factors: 38% had 1 risk factor, 22% had 2 risk factors, and 6% had 3 risk factors. These risk factors were present in 58% of the total TRAILS population. The remaining 34.0% of the subsample had none of the risk factors, and thus represented the low-risk participants. Absence of all risk factors was true for 42% of the total TRAILS population. Since both low and high risk participants were randomly selected from the TRAILS sample, the focus sample still represented the whole range of problems, e.g. the scores on all FSS items ranged from 0 to the maximum of 2. Sampling weights were used to adjust our analyses for oversampling of high risk adolescents, so adolescents without any of the risk factors had highest sampling weights (1.25) and adolescents with three risk factors lowest sampling weights (0.66).

### Procedure

#### General procedure

**Fig 1:**
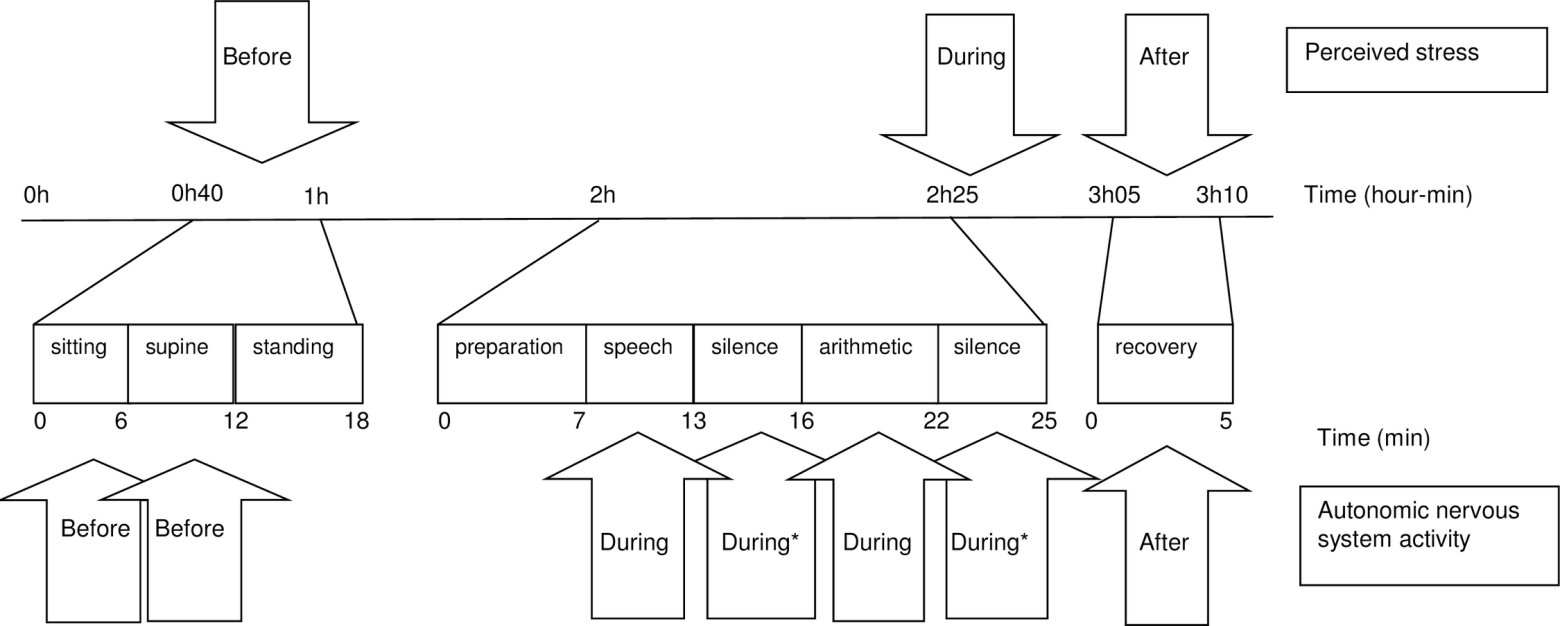
Timeline of the stress experiment and time points at which perceived stress and autonomic nervous system activity have been assessed. *used as robustness check.

The experimental session consisted of a number of different challenges, i.e. a spatial orienting task, a gambling task, a startle reflex task, and a social stress test. The session was preceded and followed by a 40-minute period of rest (Fig 1). Before, during, and after the experimental session, extensively trained test assistants assessed cardiovascular measures and perceived stress. The cardiovascular electrocardiogram (ECG) and impedance cardiogram (ICG) recordings were performed in sitting, supine, and standing position at the start of the experiment, and in sitting position during and after the social stress test, in seven blocks (Fig 1). The experimental sessions took place in sound-proof rooms with blinded windows at selected locations in the participants’ towns of residence. The total session lasted about three-and-a-half hours, and started between 8:00 and 9:30 am (morning sessions, 50%) or between 1:00 and 2:30 pm (afternoon sessions, 50%). The allocation of participants to morning or afternoon sessions was random. The protocol was approved by the Central Committee on Research Involving Human Subjects (CCMO), The Hague, the Netherlands. All participating adolescents and their parents gave written informed consent.

#### The Groningen social stress test (GSST)

The GSST was the last challenge of the experimental session. It involved a standardized protocol, inspired by (but not identical to) the Trier Social Stress Test [25]. The participants were instructed to prepare a 6-minute speech about themselves and their lives and deliver this speech in front of a video camera. They were told that their videotaped performance would be judged on content of speech as well as on use of voice and posture, and ranked by a panel of peers after the experiment. The participants had to speak continuously for the whole period of 6 minutes. The test assistant watched the performance critically, and showed no empathy or encouragement. The speech was followed by a 3-minute interlude in which the participants were not allowed to speak. During this interval, which was included to assess ANS activity measures not affected by speech, the participants were told that they had to wait for a moment because of computer problems, but that the task would continue as soon as these problems were solved. Subsequently, the participants were instructed to repeatedly subtract the number 17 from a larger sum, starting with 13,278. A sense of uncontrollability was induced by repeated negative feedback from the test assistant. The amount of negative feedback provided was independent of test performance. The mental arithmetic challenge lasted for 6 minutes, again followed by a 3-minute period of silence, after which the participants were debriefed about the experiment.

### Measures

#### Autonomic nervous system responses

A three-lead ECG and a four-lead ICG (see [26]) was registered using 3M/RedDot Ag/AgCl electrodes (type 2255, 3M Health Care, Neuss, Germany), while the participant was breathing spontaneously. Two cardiac autonomic measures were derived from the ECG and ICG data: heart rate variability (HRV), and the pre-ejection period (PEP). The cardiovascular signals were amplified with a BIOPAC Amplifier-System (MP100, Goleta, CA), and filtered before digitization at 250 Hz. ECG data were cleaned with dedicated software (i.e., PreCARSPAN,[27]) to detect R-peaks, to check signal stationarity, to correct for artifacts, and to calculate the interbeat-interval (IBI) between two heartbeats. The ECG signal was considered invalid if they contained artifacts with duration of more than 5 seconds, if the total artifact duration was more than 10% of the registration, or if the block length was less than 100 seconds. This resulted in invalid data for 38 participants before, 43 participants during, and 28 participants after the GSST. These participants were excluded from analyses that concerned these measures. Calculation of the HRV was performed by power spectral analysis in the CARSPAN software program [28,29]. HRV-HF was defined as the power in the high-frequency (0.15–0.40 Hz) band, which is associated with the respiratory cycle, and expressed in ms^2^. ICG data were assessed with a BIOPAC NICO100C Noninvasive Cardiac Output Module. The PEP reflects the time interval between the onset of the electromechanical systole (Q-wave onset) in the ECG and the opening of the aortic valves co-occurring with the B-point in the ICG. B-points were manually scored by extensively trained raters using the VU-AMS interactive software (www.vu-ams.nl), which graphically displays the large-scale ensemble averages ICG [22] over each measurement block. The ICG scoring principles given in the VU-AMS manual (2013) were followed [30]. When there was doubt about the B-point, the scoring was discussed with a second rater. Outliers were checked and quality of the PEP rates was subjectively scored on a 0–10 scale. PEP data were considered invalid if the quality of the PEP was low (i.e. a score <6) or the signal contained too many artifacts (including participants with arrhythmias or extrasystoles), resulting in invalid PEP data for 148 participants before; 163 participants during; and 160 participants after the GSST. These participants were excluded from analyses that concerned these measures. PEP is expressed in ms.

An overview of the cardiovascular ECG and ICG recordings during the experiment is given in Fig 1. The calculation of ANS activity during stress was based on cardiovascular recordings during the first 3 minutes of the speech and mental arithmetic tasks (Fig 1). Adolescents spoke during these tasks, and speech is known to interfere with analysis of HRV-HF and PEP (e.g. [31] or [32]). Therefore, as a robustness check, apart from the analyses with the cardiovascular recordings during the first 3 minutes of the speech and mental arithmetic task, the 3 minute recordings directly following the speech and mental arithmetic task were used (Fig 1). Participants were not allowed to speak during the latter recordings.

#### Perceived stress responses

Feelings of stress were assessed by means of the Self-Assessment Manikin, a non-verbal pictorial assessment technique to measure person’s psychological reactions to a stressor[33]. The subjective intensity of the feelings of arousal and unpleasantness could be indicated by choosing one out of nine ordered pictures. The pictures were recoded into a nine-point scale (range 1–9) in such a way that high scores represented high levels of arousal and unpleasantness. Feelings of arousal and unpleasantness were assessed before, during and after the stress test (Fig 1).

#### Functional somatic symptoms

FSS were assessed with seven items of the Somatic Complaints subscale of the Youth Self-Report (YSR): pain, headache, stomachache, nausea, vomiting, dizziness, and fatigue [34,35]. The items refer to complaints without a known medical cause or without an obvious reason in the past six months. Participants indicated whether they experienced these complaints ‘never’ (0), ‘sometimes or a bit’ (1), or ‘often or a lot’ (2). The scale score represents the mean of these items (range 0–2). The YSR was assessed as part of the regular T3 measurements on average 3.1 month (SD = 5.1) before the GSST, due to practical reasons.

#### Symptom clusters

In line with our previous study [20], symptoms were divided into two clusters, one consisting of headache and gastrointestinal symptoms, and the other of overtiredness, dizziness and musculoskeletal pain. All these symptoms were assessed with the YSR, apart from musculoskeletal pain, which was assessed with a by TRAILS developed pain questionnaire. We rescaled this item to the YSR. Mean item scores, which could range from 0–2, were computed for each cluster.

#### Other variables

The covariates sex, depressive symptoms, body mass index (BMI), smoking, physical activity level, medication use, which are known to be potential confounders in the relationship between ANS measures and FSS [11], were used in this study. Depressive symptoms were measured using the mean item score of 13 depressive symptoms items from the YSR (Cronbach’s alpha = .75, see [36]). BMI was defined as the weight in kilograms divided by the length in meters squared, which were measured by trained test assistants. Physical activity level and smoking frequency were assessed by questionnaire as part of the regular T3 measurements. Physical activity level was operationalized as the number of days of a week an adolescent was physically active for at least one hour. Smoking was defined as being a daily smoker (yes or no).The use of medication was assessed by means of a checklist on current medication use administered at the beginning of the stress experiment. We adjusted for psychopharmaca and sympathicomimetic drugs, since these drugs are associated with both ANS measures and FSS [11].

#### Statistical analyses

All analyses were performed while using sampling weights to correct for the oversampling on adolescents with a high risk of mental health problems. First, linear regression analyses were used to examine whether FSS were related to perceived stress before, during or after the stress test. These analyses were adjusted for sex. Linear regression analyses were also performed to examine whether FSS were related to HRV-HF or PEP before, during or after the social stress test. These analyses were adjusted for the potential confounders sex, medication use, smoking, BMI, physical activity level, and depressive symptoms [11]. Further, explorative analyses were performed to examine whether the specific clusters of FSS were differentially related to perceived stress levels and HRV-HF or PEP levels. These analyses were additionally adjusted for the other cluster, since the clusters of FSS were simultaneously included as predictors in the model. Finally, cardiac ANS reactivity was examined. In line with previous research [37], ANS reactivity measures were determined by the maximum HF-HRV level during the test minus the minimum level before or after the test (ΔHF-HRV) and the maximum PEP level during the test minus the minimum level before or after the test (ΔPEP). Linear regression analyses were performed to examine whether these ANS reactivity measures were related to FSS. Analyses were performed in SPSS version 22. The database used to perform our analyses can be found in S1 Dataset. Results were considered statistically significant if p <0.05.

## Results

### Descriptive Statistics

Information on age of the participants, sex, FSS, perceived stress levels, ANS activity and covariates are shown in Table 1. Mean item scores of the FSS clusters were comparable (Table 1).

**Table 1:** Sample characteristics.

	Valid *N*	Mean (*SD*) or percentage
**Female**	715	50.9%
**Age**	715	16.1 (0.60)
**Body mass index**	696	21.3 (3.28)
**Depressive symptoms**^a^	695	0.25 (0.24)
**Medication use**^b^	715	4.3%
**Smoking (daily)**	699	17.3%
**Physical activity level**^c^	696	3.3 (2.1)
**Functional somatic symptoms**^a^	675	0.34 (0.33)
**Cluster of gastrointestinal symptoms and headache**^a^	680	0.39 (0.43)
**Cluster of overtiredness, dizziness and musculoskeletal pain**^a^	679	0.35 (0.36)
**LN HRV-HF before**^d^	677	6.7 (1.2)
**LN HRV-HF during**^d^	672	7.0 (1.0)
**LN HRV-HF after**^d^	687	7.0 (1.1)
**PEP before**^e^	567	125 (19)
**PEP during**^e^	552	111 (19)
**PEP after**^e^	555	123 (19)
**Perceived arousal before**^f^	704	2.6 (1.5)
**Perceived arousal during**^f^	713	4.2 (1.9)
**Perceived arousal after**^f^	697	2.4 (1.5)
**Perceived unpleasantness before**^f^	704	3.2 (1.5)
**Perceived unpleasantness during**^f^	713	4.8 (1.8)
**Perceived unpleasantness after**^f^	697	2.9 (1.8)

^a^mean item score which could range from 0–2;

^b^use of psychopharmaca and sympathicomimetics drugs;

^c^mean number of days a week on which at least one hour physical active;

^*d*^*LN = natural logarithmic transform;*

^e^ln(ms^2^)

^*f*^*mean item score that could range from* 1–9; *before*, *during and after refer to the Groningen Social Stress Test (GSST)*

### Perceived Stress Levels Before, During, and After the GSST and FSS

FSS were positively related to perceived arousal levels before, and during the GSST and to unpleasantness levels before and after the GSST (Table 2). Explorative linear regression analyses revealed that these associations were symptom-specific. The cluster of headache and gastrointestinal symptoms was only positively related to perceived unpleasantness during the GSST (Table 2). The cluster of overtiredness, dizziness and musculoskeletal pain was positively related to perceived arousal level before, during and after the GSST and to perceived unpleasantness before the GSST (Table 2).

**Table 2:** The associations between perceived stress before, during, and after the Groningen Social Stress Test (GSST) task and FSS.

	Perceived arousal before	Perceived arousal during	Perceived arousal after	Perceived unpleasantness before	Perceived unpleasantness during	Perceived unpleasantness after
**Functional somatic symptoms**	0.06 (0.11)	0.09 (0.01)*	0.07 (0.047)*	0.07 (0.048)*	0.12 (0.001)**	0.06 (0.10)
**Cluster of headache and gastrointestinal symptoms**	-0.01 (0.76)	0.05 (0.23)	-0.06 (0.17)	-0.02 (0.62)	0.09 (0.03)*	0.04 (0.42)
**Cluster of overtiredness, dizziness and musculoskeletal pain**	0.09 (0.043)*	0.10 (0.02)*	0.16 (<0.001)**	0.12 (0.005)*	0.07 (0.10)	0.04 (0.40)

Beta (p-value); all analyses are adjusted for sex and medication use; the two symptom clusters were analyzed simultaneously and thus effects were adjusted for each other; before, during and after refer to the Groningen Social Stress Test (GSST)

*p<0.05

**p<0.01; Note: sampling weights were used to represent the distribution in the normal population (see method section for more details)

### Cardiac ANS Activity Before, During and After the Stress Experiment and FSS

Neither HRV-HF nor PEP measures assessed before, during or after the GSST were significantly related to FSS (Table 3), nor were ANS activity measures related to specific FSS clusters (Table 3). As a robustness check, we repeated these analyses using the interludes directly after the speaking and arithmetic tasks, during which participants were not allowed to speak to avoid the influence of speaking on autonomic measures. HRV-HF and PEP assessed during these interludes were not significantly related to FSS in general or to specific FSS clusters. When examining ANS reactivity (i.e., ΔHF-HRV and ΔPEP), HRV-HF reactivity (beta = 0.03, p = 0.58) and PEP reactivity (beta = -0.05, p = 0.39) were not related to FSS.

**Table 3:** The associations between ANS activity before, during and after the Groningen Social Stress Test (GSST) and FSS assessed in sitting position.

	HRV-HF Before	HRV-HF During	HRV-HF After	PEP Before	PEP During	PEP After
**Functional somatic symptoms**	0.02 (0.63)	-0.004 (0.94)	0.02 (0.65)	0.05 (0.32)	0.07 (0.19)	0.04 (0.47)
**Headache and gastrointestinal symptoms**	0.001 (0.99)	-0.06 (0.17)	0.03 (0.48)	-0.04 (0.46)	0.000 (1.00)	-0.04 (0.47)
**Overtiredness, dizziness and musculoskeletal pain**	0.01 (0.80)	0.06 (0.25)	-0.03 (0.64)	0.11 (0.050)	0.07 (0.25)	0.07 (0.21)

Β (p-value); All analyses are adjusted for sex, medication use, smoking, exercise frequency, body mass index, and depressive symptoms; the two symptom clusters were analysed simultaneously and thus effects were adjusted for each other. LN = natural logarithmic transformed; HRV-HF = heart rate variability in the high frequency band in ln(ms^2^); PEP = pre-ejection period in ms; before, during and after refer to the Groningen Social Stress Test (GSST); during refers to the averaged cardiac measures assessed during the first 3 minutes of the public speaking and the mental arithmetic task; Note: sampling weights were used to represent the distribution in the normal population

## Discussion

This study in 715 adolescents from the Dutch general population revealed that perceived arousal levels during and after the GSST and perceived unpleasantness levels before and during the GSST are related to FSS experienced in the past couple of months. These associations were specifically evident in the cluster of overtiredness, dizziness and musculoskeletal pain in contrast to the cluster of headache and gastrointestinal symptoms. Cardiac PNS and SNS activity before, during and after the GSST were not related to FSS in our study, nor were cardiac PNS or SNS reactivity.

One strong point of our study is that we examined the associations between ANS functioning and FSS in a relatively large sample, which increased the robustness of our findings. Furthermore, using sampling weights enabled to demonstrate that findings could be generalized to the general population of adolescents. Moreover, the assessment of a wide range of variables enabled us to adjust for many potential confounders. A final strength is that perceived stress and cardiac ANS measures were repeatedly assessed before, during, and after the GSST. This allowed us to show that FSS were stronger related to perceived stress during than perceived stress before or after the GSST.

One limitation of our study is that the ANS activity measures assessed during the stress test performance might have been disturbed by speech. As a robustness check, the stress levels during the interludes, when adolescents were not allowed to speak, were used. Stress levels remained relatively high during the interludes, because the participants expected that they had to continue any moment. Nevertheless, they were lower than during speech and mental arithmetic performance. These problems regarding speech might have weakened the potential association between ANS activity and FSS. Another limitation is that stress responses were defined in relation to either pre or posttest levels, depending on whether stress levels were lower or higher before or after the test, instead of only pretest levels. This approach was taken since it allowed us to study maximal stress responses, and was in line with previous TRAILS research. Further, the on average 3.1 month delay between FSS assessment and the stressor task may have diminished the power to find potential associations, since adolescents’ FSS level could have changed in the meantime. Furthermore, it should be noted that the FSS scores in our study were quite low, which might have accounted for the lack of association between cardiac ANS measures and FSS. Cardiac ANS functioning might be disturbed in adolescents with severe FSS, which was only a small proportion of our sample. Finally, it should be noted that the significant associations found were between two self-reported measures (i.e. FSS and perceived arousal/unpleasantness). Although the measures were assessed at different time points, we are not able to rule out that the found associations are partly due to report bias.

In keeping with previous research, this study showed that FSS are positively related to perceived stress levels during a stressful situation [5–7]. In addition to previous research, it shows that the nature of this association depends on the investigated symptom cluster. The cluster of headache and gastrointestinal symptoms was only related to perceived unpleasantness during the stress part of the GSST, which might, taking multiple test bias into account, be considered a chance finding. The cluster of overtiredness, dizziness and musculoskeletal pain was much stronger related to perceived stress than the cluster of headache and gastrointestinal symptoms, and showed association with perceived stress before, during and after the GSST. Therefore, this cluster seems associated with anticipation stress, high stress reactions, and psychological recovery problems after a stressful situation occurred. These findings thus suggest that this symptom cluster is stronger related to psychological disturbances than the cluster of headache and gastrointestinal symptoms.

In line with some previous studies, HRV-HF and PEP were not related to FSS [10–18]. This study did thus not find evidence that the potential association between cardiac ANS functioning and FSS is particularly evident during stress. This might bring into question whether it is not the other way around: that an association between ANS functioning and FSS becomes particularly evident during rest. Adolescents suffering from FSS might especially differ from healthy subjects in that they are not able to relax during a non-stressful period. Such a hypothesis is supported by the fact that we did find associations between HR-HRV and FSS in supine position at rest in adolescents and young adults in former studies, in which the rest assessment did not precede a stress experiment and when participants therefore probably felt more relaxed [10,12]. However, differences between this and our two former studies might also be explained by the sample size that was larger in the former studies, and therefore previous studies might have had more power to show significant effects.

Since we found subjective stress (i.e., arousal and unpleasantness) to be related to FSS in adolescents, diminishing subjective levels of arousal and unpleasantness in adolescents might be a good intervention target for FSS. This is in line with the few studies that examined the effectiveness of stress reduction in adolescents suffering from FSS (e.g. [38] and [39]). Regarding the biological background of the association between subjective stress and FSS, we did not find evidence for a role of the cardiac ANS. This might have to do with the fact that the ANS is mainly involved in short-term stress responses. We previously found some evidence that the long-term stress regulation system, i.e. the hypothalamic-pituitary-adrenal-axis (HPA axis), is related to FSS [20].

In conclusion, the findings of this study suggest that perceived stress levels before, during, and after a stressful situation are related to FSS in the past couple of months. This was specifically true for symptoms of overtiredness, dizziness and musculoskeletal pain in contrast to headache and gastrointestinal symptoms. Cardiac ANS activity levels before, during and after a stressful situation were not related to FSS in our study, nor were HRV-HF and PEP reactivity.

## Supporting Information

S1 Dataset Dataset for the analyses. (XLSX)

## Abbreviations

ANS: autonomic nervous system
PEP: pre-ejection period
HRV-HF: heart rate variability in the high frequency band
ICG: impedance cardiogram
ECG: cardiovascular electrocardiogram
BMI: body mass index
